# 50 years a biomedical engineer remembering a long and fascinating journey

**DOI:** 10.1186/1475-925X-11-1

**Published:** 2012-01-01

**Authors:** Max E Valentinuzzi

**Affiliations:** 1 Instituto de Ingeniería Biomédica (IIBM), Facultad de Ingeniería (FI) Universidad de Buenos Aires (UBA) Paseo Colón 850, (1063) City of Buenos Aires, Argentina

## Abstract

Looking back at one point of life appears as a nice exercise to round out and summarize. However, the objective should not be simply to tell a story; it must transmit a message to the young. To start with, two concepts are useful: *Respect for others begins when you learn to laugh at yourself *and, taken from an old saying, *I did not want to be poor ... but money wouldn't make me rich*. After elementary and high schools, during times of turmoil, I describe my engineering school years at the University of Buenos Aires and a working experience in an international telecommunications company. Significant events taught me a concept, rooted in another motto: *Isn't this house nice? It is my house, and I love it very much*. In 1960, I began my activities in the USA. A couple of bad decisions resulted in significant events for me teaching me an important truth: "Beware of golden promises; time is the most precious asset". Finally, in 1972, settled down in Tucumán until retirement in 2001, a long period of productive activity came about, not without difficulties and also stained by a dark political interval. Crises seem to characterize our generations in Argentina. Non-the-less, there were some real accomplishments: an undergraduate program in BME and a National BME Society (SABI) plus an archive of specialized published material. After spending time following retirement in Peru and Italy, my current activity came as unexpected dessert at the University of Buenos Aires, with a small research group, so offering the opportunity of transmitting what I still have available.

## Looking back ... nice exercise

Life is like a play, where each of us is in the star role, or at least that is what we think. Many scenes show up sequentially, mixing different events; many vanish inconsequentially, while a few strongly mark our road and remain as indeleble hallmarks. The bad are often forgotten ... when possible. At one point, there is a tendency to look back. If such retrospection is enhanced by friends, one may end up believing that good material is at hand. An early point in this note to bring up, as a message that I did read somewhere sometime back: "*You will begin to respect others the very moment you learn to laugh at yourself*". It took me many long years to recognize this truth.

## Early years, elementary and high school

I finished the last paragraph with a thought; let me start this one by rescuing another, forgotten by me at first and, thereafter, coming back to stay in my memory to and, somehow, unconsciously signalling my life. It is an old German saying: *Ich wollte nicht arm sein ... aber Geld würde mich nicht reich machen (I didn't want to be poor ... but money wouldn't make me rich)*. It transmits a message to be at least briefly considered by the young, since always there is time to discard it if not found relevant.

At age 3, in Buenos Aires City, I started kindergarten (German word literally meaning "a garden for children") in the *Germania Schule*, and for two years thereafter, 1st and 2nd grade at the *Humboldt Schule*. In 1940, I was placed in 3rd grade, again in the *Germania*, finishing there the elementary level of my education. It is significant to call attention to those full 10 years: 1935-1944, the Nazi period and the 2nd World War (WWII). I well recall Hitler's picture on the back wall of every classroom. The German community was rather large in Argentina; it maintained several schools, all associated to the *Deutsche Schulverein *(German Schools Association). Annually, around October, it organized an excellent sports competition held in Vicente López, a northern and cozy suburban area by the River Plate. One of the outstanding activities was *völkerball *(people's ball), which I liked very much, being a good and often acclaimed player in spite of my small size. Overall and in retrospectroscope, yes, the education given was demanding and of excellence, covering a wide and in-depth spectrum: history (Argentine and world), geography (Argentine and world), languages (including their grammars and orthography), arithmetic, geometry, sciences (biology, physics, chemistry), music (my first contact with it, learning to play the sweet flute), manual activities (painting, drawing, ceramics), gymnastics (strong and good program).

When I was 10, my father bought a second-hand piano (a THEIN, German made; from its serial number engraved on the inner right corner, I learned much later that it was built in 1912). I still have it and use it daily. Music became part of me ever since and, already in high-school, since one of my classmates was Lalo Schiffrin, now an internationally well regarded Argentine-American pianist and composer (remember *Mission Impossible*?), one day he came to my house with other classmates, all being 15 or 16, and he played for a while on that piano. Deeply impressed I was left by his extraordinary ability and even dreaming to some day play like him (which obviously did not happen because, unfortunately, I was not a good student and, more important, I did not enjoy a natural musical gift). That is why I always claim that my best music curriculum is that Lalo Schiffrin played in my piano.

In 1945, when the war was over, all the activity of the German schools essentially disappeared by a government decree. However, the German community re-emerged in the country after WWII and still constitutes a significant and influential part of the Argentine population. Argentina owes many contributions to these people [1]. Let us not become polarized by prejudices simply based on historical sad events and personal experiences.

Everything in these German schools was within the frame of a rather military-like system, including corporal punishments (usually heavy buttock spanks with a ruler or slaps on the face). Many teachers were frankly pro-nazi, especially manifested during the two or three years before closure, when several "new teachers" not knowing a single word of Spanish showed up; obviously, they were refugees fleeing the crumbling crazy regime. How unhappy I felt. Several times, I begged my father to remove me from that school but he would not even consider the possibility. My father, a medical doctor who later in life got a degree in physics and mathematics, was a democrat from his inner guts, a socialist by conviction, although having never participated in any political activity. He also used to repeatedly mention the utopian concept of **Republic of the World**, once dreamt of by a few idealists. Obviously, he did not accept and greatly deplored the nazi ideology and its cruel acts, but he admired the German culture at large: literature, philosophy, music, science ... and frequently used to mention in our almost daily family talks Goethe, Schiller, Bach, Mozart, Beethoven, Kant, Nietsche, its great physicists ... He read German fluently and spoke it relatively well, all learned by himself. No doubt, his eldest son had to be intellectually formed within that context!

But the generation of germanic antibodies within me grew steadily and stronger as time went by, and I began to reject the language (which at one time I had acceptably mastered; I still have good phonetics and accent). My behavior became really bad and I think I was not expelled because of the political situation in all Argentine German Schools. Due to the international conflict, those schools had entered into a dead-end and were to disappear while the Mother Country was headed to almost complete destruction and rebirth anew. On two occasions, one very early (I was 6 or 7), a lady teacher, *Fräulein *Margueritte Heisecker, undeservedly slapped me hard in front of the full class (probably, she was confused by one of those children's messes). My reaction was terrible: I grabbed her by the skirt and started to kick her ankles like crazy, thereafter running out of the room all around school and trying to reach the street. My father was called and somehow the situation was solved. The second time was worse by far. I was 12, already in the last grade, the 7th. It was the second half of 1944, everybody knew Germany was falling to pieces, and at school the atmosphere was tense. New unknown faces had appeared and my behavior was getting worse. One day, *Herr *Kasis (who incredibly looked very much like Hitler, his hairdo and moustache), slapped me very hard after one of my obvious misconducts. I fought back, punching him all over while he responded even harder hitting me on my face and head and pushing me uncontrollably out of the room. Once more my father was urgently called while they could not find me because I hid in the gymnasium. Finally, in the principal's office (*Frau *Winzer, a tall, bitter and ugly lady, perhaps in her forties), with my father and several other teachers present, I yelling madly all sort of insults, calling them dirty nazis SOBs and my father threatening to go to the Ministry of Education pressing charges of overt child mistreatment, the whole thing quieted down and less than two months later I finished school with an undescribable feeling of freedom. On December 9, 1944, I took the entrance examination to the *Colegio Nacional de Buenos Aires*, of the University of Buenos Aires [2].

However, my "revenge" was still simmering and I decided to actively forget all the German I knew, which I punctiliously and succesfully accomplished in record time. Can you imagine the size of my stupidity? Do you, reader, know how many times in my life I have needed that language? Do you know how I suffered during a full month in Vienna in February 1989? Years later, my father and I were in a small town of North Carolina waiting for a bus to Cullowhee, where a Biomathematics meeting was to be held, and we started to talk about my German school experience: He admitted he should have taken me out of that school and I humbly and embarrased too, confessed with great regret my childish constant bad behavior and subsequent revenge. The German I keep is residual while reading means a lot of effort followed by a painful headache! My father had to leave Argentina after the 1955 revolution (he was accused of having collaborated with the peronists ... he, with the ideas about freedom, *Freiheit*, he sustained!) and settled down in Chicago, where he worked at that university with Nicholas Rashevsky and collaborators in his notable Committee of Mathematical Biology. Later he became an American citizen.

The six years at the University of Buenos Aires high school (1945-1950) left an enduring imprint, on one hand for the then existing educational system strongly based on a long tradition of democracy and academic freedom encompassed within strict discipline and mutual respect, and on the other hand marked by the political times inaugurated by Juan Domingo Perón, who essentially took control of the country between 1945 and 1955. By and large, students opposed Perón in those days and the *Colegio Nacional de Buenos Aires *(CNBA) was no exception, frequently participating in public manifestations that often ended violently. During one of those events, I was part of a fight and, along with a companion, we were taken to the near police station and held there for several hours. Oh, my mother ... when finally they let me call her late in the night! She had to pick me up and sign some document that allowed my release.

An interesting side comment is pertinent. At 14, I started having athsma spells accompanied often by heavy colds or even flu, a mixture of allergy and sometimes perhaps bacterial or viral infection. They really disturbed my student activities. Since I had become a member of the University Club of Buenos Aires (CUBA, or *Club Universitario de Buenos Aires*, in Spanish, not connected to the university itself except that all its members must be university people, either students or professionals or professors), one day I decided to attend the gym classes of respiratory exercises, offered by Carmelo Robledo, who had been Olympic Box Champion during the 1932 Los Angeles Games, in the Feather Category (57-58 kg of body weight). However, those classes were attached to fighting, too, and there I became by fact an amateur fighter, even being part of a few internal games (where once I won a medal while in another occasion a forbidden head hit from my opponent produced a good eyebrow wound and heavy bleeding). Those respiratory exercises did some good to the athsma problem and I kept punching back and forth three times a week, until one day, already in the engineering school, the boxing professor invited me to the annual dinner held by the amateur federation of boxers, incidentally near my home. What I saw that evening I did not like at all; many relatively young guys acting as mentally disabled or retarded people ... and I quickly dropped out of the classes and the boxing group. Carmelo Robledo himself, years later, died of a cerebrovascular accident (CVA). At 28, after I settled down in Atlanta, GA, athsma magically vanished and never bothered me again. Was it the dry weather of that region as opposed to the humidity so frequent in Buenos Aires? Peter Kellaway, a neurophysiology specialist and former professor at Baylor College of Medicine, I recall well during my graduate period there, gave us a couple of lectures about concussion and he spoke of boxers and the micro-hemorrhages produced by the many hits on the face and head. Their effects are cumulative, often showing electroencephalographic (EEG) changes that may last for two or three months and, in cases, may never disappear, so explaining the abnormal behavior of many former fighters and their eventual CVA's. Advise: do not practice boxing because that is not a sport. Wise was my decision to quit it after that dinner!

Programs and syllabi at the CNBA were excellent, perhaps too biased toward humanism and somewhat encyclopedic in their contents, but there is no regret at all because the background we got was solid and longlasting. French was mandatory for everybody the first four years, with the choice at the fourth of adding either English or German, which continued until graduation. Well ... I took the former pushing the latter as far away from me as I could (still entrapped in the revenge spell). Besides, we had six years of Latin, but its success was poor, at least with me; however, French and English well penetrated the brain barrier and, full of glee, many years later I was able to move softly and smoothly in the USA, France, Holland, Canada, India and Israel. We had courses of Spanish literature (in which the Cervantes' Don Quixote stood out significantly, in fact, a little too much), philosophy, history and geography in detail, a good deal of sciences. A beautiful covered swimming pool near a large basement gymnasium within the main building, complemented with an outdoors sports field located in the nearby city harbour area, gave us the opportunity of burning our super-abundant teenager energies. I enjoyed that adolescent period of my life, even winning an internal paid fellowship that made me a proctor for a full year (1950), along with the distinction of being placed in the school frame of honor. How different it was from the previous elementary school period. Proud I was, overflowing with drive to do things. The question most of us faced was how and where to proceed to the third stage of our education.

## Engineering school at UBA

**Figure 1:**
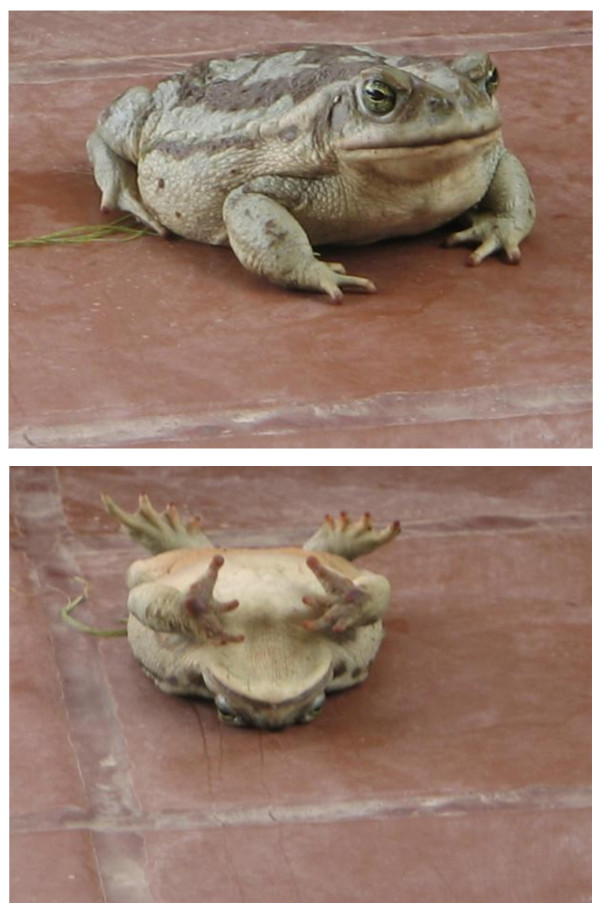
**The aesthetic beauty of a frog**. Beautiful adult specimen of a *Bufo arenarum*, common frog (actually a toad) found in Argentina, especially in the Northwest, which we used frequently in our experimental exercises. Unfortunately, it is disappearing because of human carelessness. Below, the same animal after being "hypnotized" by gently scratching its belly (a typical response, mostly seen in the male). Students used to look with surprised eyes when I run this little demonstration while calling the girls' attention to the probability of being a bewitched prinz. Isn't it a beauty, doesn't it possess aesthetic content? Look at its gleaming eyes, besides, they are harmless ... and they can quickly catch a flying fly or mosquito. In Houston, Dr. Hoff used to make the same demonstration, to illustrate the humanitarian use of animals in the labs and also as an introduction to neurophysiology. Picture taken by MEV at his home, in Yerba Buena, Tucumán, when explaining this behavior to his grandson, Max IV (6 years old).

Since the *Colegio Nacional de Buenos Aires *belongs to the *University of Buenos Aires *(UBA), it takes 6 years to graduate instead of 5, as other schools do. Its programs differ significantly, no entrance examination is required to be admitted to any of the third educational level faculties (colleges or schools). For a long time, I was considering the idea of a career in biology or perhaps medicine or even chemistry. What influenced me on these possible choices? The first two are clearly rooted on my father. The last two or three years of my elementary school years (1941-4) were very frequently seasoned by visits to the Laboratory of Biophysics my father headed, which was in the basement of the Argentine Academy of Medicine (*Academia Nacional de Medicina*), housed in a beautiful building still existing and active, at the corner of Las Heras Avenue and Coronel Díaz Street, a nice area of the city of Buenos Aires, near the traditional Palermo Park. It was there where I saw for the first time a beating frog's heart, where I learned how to operate a Poggendorf potentiometer while my father explained me the nerve and muscle action potentials in simple terms and the concept of the electric and magnetic fields. More than that, constantly my father, as gynecologist and obstetrician, insisted on the respect for life and love to animals at large. No doubt, they were seeds that stayed deep in me [3-9]. In high school, the chemistry profesor, Dr. Antola, not really too kind, but dedicated and efficient in his lectures, both theoretical and in the laboratory, lighted a flame in me. Also in high school, Prof. Antonio Valeiras was highly motivational in his math clases. One remark he once made, after a long demonstration on the blackboard and asking the class to keep silent for a minute while looking at it, impressed me deeply. He said: "*Is that not beautiful*?" Many times thereafter in my life I repeated such comment to my students, disciples or collaborators: "*Isn't that experiment, that surgical field, that graph, that formula a beauty*?" To pursue and find the aesthetic content and leads to true freedom [10], as Friedrich Schiller (in *An die Freude*, to joy, even though he meant *Freiheit*, liberty) and Ludwig van Beethoven (in his 9th Symphony) proposed, represents an essential mission of a human being, perhaps what makes him/her human and different from animals. Theirs was an eternal message to all mankind. We may slightly modify the thought as "***the aesthetic content leads to true freedom and richness of spirit***." Look at the frog shown in Figure 1, for example, isn't it a beauty, isn't it aesthetic?

However, the decisive turning point took place at the end of 1950, when Dr. Jean Charles Vignaux, professor of mathematics at the UBA and at the Military Naval School, invited a group of final year high school students to visit the Institute of Radiotechnique. There we went, without knowing exactly what the school was all about. He was the director, an extremely kind man in his 60's, who accompanied us all morning, showing each laboratory, explaining everything, introducing us to several of the scientists working there (some had been hired in Germany), showing us oscilloscopes, oscillators, receivers and transmitters, antennas, and a bunch of other electronic equipment. We left with our eyes wide open and our cerebral neurons tickling: "*Boy, that's for me, I want to become a Radio Engineer*", for that was the name of the brand new career.

Unfortunately, the political scene had worsened, and universities and their people were not looked upon with favorable eyes in some environments, in some areas they even were openly unwelcome. Many professors left the country and several laboratories closed [11]. Non-the-less, somehow the new career proceeded more or less untouched. At one point, its name was changed to Telecommunications Engineering, and that is what the diploma given to me in 1956 says: ***Telecommunications Engineer***, perhaps equivalent to a bachelor plus or a master minus on the American scale. Perón, meanwhile, was ousted by a rather cruel revolution in September 1955, giving way to a refreshing new stage for the universities; however, a huge mistake of that revolution was to ban the peronist party and any manifestation favoring it.

## Short pulses in time multiplex communicating cities

The job market was eager of young graduates and all Telecommunications Engineers could choose among several offers. In my case, and a few months before graduation, I started with a private telecommunications company, TRANSRADIO INTERNATIONAL, subsidiary of RCA Communications of New York. Soon thereafter I was offered positions in the National Ministry of Health, the Atomic Energy Commission, a private company that was installing a microwave system to monitor an oilduct in the northwest region of the country and the Military Navy, but I decided to remain where I was, and it was good, indeed. What came after did not disappoint me at all. I worked, studied, designed, learned, interacted with many people and made my first practical use of spoken English. Five full years I spent there (1955-1960), reaching a managing level after passing through its main sections: the trasmitting and the receiving stations (Montegrande and Villa Elisa, respectively, both located in suburban areas), the central office facilities and the design laboratory, the two latter placed downtown Buenos Aires. I even enjoyed a simultaneous period at the University of Buenos Aires that I finally had to resign because the time requirements became difficult to satisfy. Incidentally, my interview at TRANSRADIO took place on September 16, 1955, a Friday, the day the so called *Revolución Libertadora *mentioned above broke off; right across the street (San Martín and Corrientes Ave, in Buenos Aires City), there was a building occupied by the *Alianza Libertadora Nacionalista*, a pro-nazi dark organization that a few days later was blown up with heavy weapons by the revolutionary army. I recall armed men wearing bracelets mounting guard on the sidewalk. My first day of work was the following Monday, September 19, in the Transmitting Station located in Montegrande (behind the Ezeiza International Airport). After a two hour bus trip very early in the morning, under heavy rain, I crossed a flooded antenna field to reach the main building. The boss in charge, Mr. Héctor Peña, could not believe I had made it. Meanwhile, the revolution kept going on with fightings in Córdoba City, in the Province of Entre Ríos and in Buenos Aires City. The death toll was significant. Something sad to remember ...

At the TRANSRADIO Transmitting Station I was a technician. When I graduated, I was transferred to the Central Office, at the San Martín and Corrientes Avenue building mentioned above. That was in July 1956, but my last day in Montegrande took place earlier, on March 23, when working with the output power amplifier of a 27 MHz transmitter, and due to a faulty ground connection, I suffered a 2,400 volt dc discharge that produced respiratory arrest (not cardiac fibrillation). People around me gave me manual artificial respiration for 45 minutes ... and lo and behold, a first deep breath came back! Still I carry the burn scars on my left hand and right arm while I contend that my second birthday is March 23, 1956 (my biological birth is on February 24, 1932), so practically making me 24 years younger. I never could imagine that in the far future fibrillation-defibrillation and resuscitation maneuvers would become one of my research subjects. Those who like the *karma *idea, which according to Vedic literature is the law of cause and effect, well, take it or leave it.

**Figure 2:**
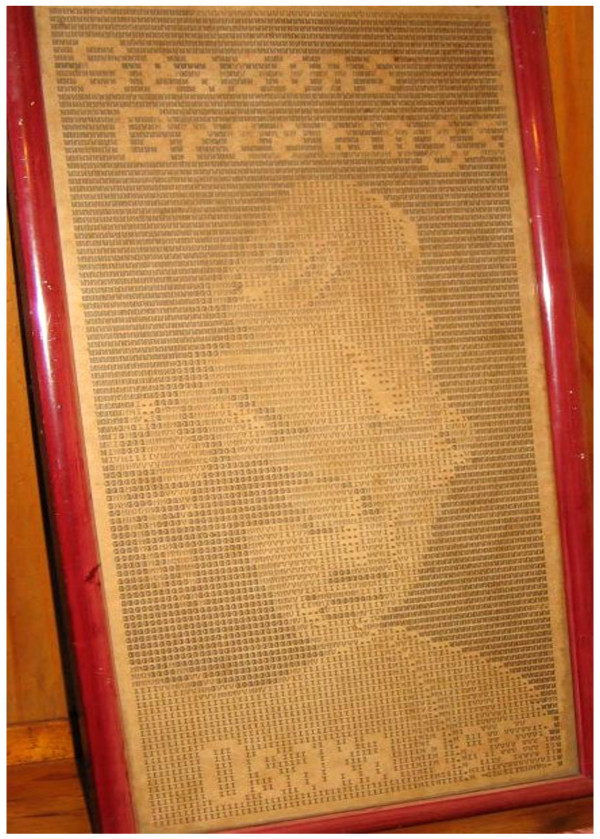
**Buda silhouette**. It was drawn with letters by a teleprinter using the TOR equipment (a time multiplex protected system) of those days transmitted from Osaka, Japan, to Buenos Aires, in December 1957, when Max Valentinuzzi was a young telecommunications engineer in TRANSRADIO INTERNATIONAL. A punched paper tape drove a synchronized electromechanical sender.

My technical experience gained there was outstanding: in transmitters and in all the equipment used for information exchange, especially the time division multiplex systems. For the first time in Argentina we installed the so called Teleprinting Over Radio (TOR), in electromechanical and electronic versions (both, vacuum tubes and transistorized), with very simple error correcting codes. We spent hours in touch with several cities around the world via teleprinter, collecting contacts and friends in New York, Tokyo, Paris, London and other European cities. I still keep framed a Buda drawn by the teleprinter that was sent one night from Osaka with the note SEASON'S GREETINGS, because the 1957 Christmas was coming close (Figure 2). Besides, I became a strong addict and fan of communications (and still am), who spoke always with pride of "*the 5 ms or 2.5 ms pulses*" crossing the oceans trying to impress my interlocutor. Those duties gave me the chance of acting for three months as assistant to Mr. Christian van Dalen, a superb Dutch engineer (both technically and as a human being), who had worked during the war years in the underground resistance developing the TOR system along with his close collaborator, Mr. van Duuren, another Dutch engineer. He played piano and came several times to my house where we dined and enjoyed playing music together. In those days, I dreamt of going abroad and he told me the following story after stressing, *yes, go wherever you consider best, but keep in mind the caveat (beware) behind this little story*,

*Once, a young man wanted to travel the world over looking for money, to become rich. So he did, spending long years an hard efforts in his search until, already tired, beaten by life, poor and rather old and sick, he had news that in his home garden a gold mine had been found. (Maybe more familiar as the Bluebird of Happiness story ... "gotta find a way back home" ... as a song lyrics says)*.

Good point to recall a traditional German epigram:

*Ist dieses Haus nicht sehr schön? Es ist mein Haus, und ich liebe es sehr!*

*(Isn't this house nice? It is my house, and I love it very much!)*

During those years I produced my first four papers [12-15], two of them related to information theory [13,15], which somehow led me to biology and medicine. I now look at these early "brain waves" with a smile and condescending fondness, also reminiscing the long study hours with my father. The journal where they appeared, *Revista Telegráfica Electrónica*, is the oldest of its kind in Argentina, founded by Domingo Arbó (Don Domingo), in 1912 (funny and dismaying, the same year the IRE was born but how different the paths), with the shorter name of *Revista Telegráfica*. Originally, it was mainly devoted to the radio-amateurs, expanding later on its reach. But, for many reasons, it never obtained much international recognition, only reaching to some Latin American countries [16]. After this, a long wavy road was yet to be run by me.

## Computers, barely getting a little feeling

As mentioned above, for slightly more than a year (1958-9), I also had an assistanship at the UBA, where one of my former professors of mathematics, the dear and highly respected, Dr. Manuel Sadosky, organized a seminar on computers. His objective was to create a group able to design and build such machines. Every week, on Thursdays at 8:00 pm sharp, we gathered to listen to a speaker, usually one of us, who had studied a specific subject. My topic on one occasion was the design of a vacuum tube flip-flop, which I took from a paper published in one of the *Institute of Radio Engineers Transactions*, or IRE (the current IEEE, Institute of Electrical and Electronics Engineers, was formed in 1963 by the merger of the IRE, founded in 1912, and the American Institute of Electrical Engineers, or AIEE, founded in 1884). We were about 30 young men, all enthusiastic and full of pep, for Sadosky knew how to inject drive. This group can be considered, perhaps, the seed of the Institute of Calculus where the first UBA computer, a Mercury-Ferranti (baptized *Clementina*), was installed a few years later. It recently celebrated its 50th anniversary, in May 2011 [17,18]. However, though a nice learning experience, for me, it was only a tangential activity that left me some good memories and some knowledge.

## Emory university and Baylor College

The colloquium referred to in [15] was another turning point in my development. Via a chain of events, the possibility of going to the USA suddenly and unexpectedly came up in 1960 and, by the end of September of that year, I set my foot in the Biophysics Lab of the Department of Anatomy at Emory University, in Atlanta, GA, entering the country as permanent resident (my wife Nilda, too). How different it was 51 years ago! Better not mention how things are now and what treatment consulate offices offer, by and large, to foreign applicants. Everybody is, *prima facie *and just in case, considered a potential undesirable. Oh, yes, there are reasons ... but ... Many visas are denied without a word of explanation. But this is a matter well beyond this essay's objectives.

My almost two years at Emory (1960 last quarter-1962 first quarter) were a pain in the neck, a true waste of time in the hands of two Argentine charlatans and even perhaps mentally cracked (especially one of them, who never got a university degree, and for the other one who was only a puppet), who pretended to be scientists. So much time has elapsed that I had better leave their names hidden in the backstage. I could never understood how they had gotten substantial grants in the USA. However, I did not stay put all that time: I studied a lot of electrophysiology, going into the most significant papers by Kenneth Cole (years later, in 1976, I met him in Ottawa), Hodgkin, Huxley, Katz and other important contributors to the field. Emory's Main Library is a beauty, where I used to spend hours digging endessly into its rich shelves. Its Alumni Building was a refuge where I used to play piano. As my superiors had put me in charge of the electron microscope (an RCA-EMU-2) and also used me as their personal calculator (no computers yet in those days), I also studied a lot of electron microscopy, its necessary photographic techniques and considerable statistical procedures for standard data analysis. All of this turned out to be very useful in the future, indeed. No regret, really. A few years later, I even published a didactic article on electron microscopy in an obscure Argentine journal [19], just to satisfy myself, perhaps trying to make up for an overt admission of failure. Meanwhile, the local situation at Emory was sliding down and my relationship with the two men above me was unbearable, especially after a rather strong argument. After a letter from my former professor (mentioned above), Dr. Manuel Sadosky, I got in touch with an MD in Houston, TX. He was organizing a symposium on information theory and the nervous system, at Baylor University College of Medicine, to be held by the end of March 1962. I needed fresh air. First, my wife Nilda and I, carrying our baby daughter Fabiana (6 months old, born at Emory Hospital in October 1961, and now a resident with her family in the USA), had driven to the Northeast (Washington DC, Princeton, New York) in a black 1951 Chevrolet looking for opportunities ... and I found some possibilities: one, in Princeton, but not very convincing, another in Brookhaven National Laboratory, Long Island, where I was lucky to be interviewed by Henry Quastler (1908-1963), outstanding Viennese physician and radiobiologist. The possibility looked stronger over there, but I lost contact with him. Later on, I learned he had committed suicide the year after I saw him. After this trip, already in the last week of March, we were again on the road, this time heading to Houston following the gulf route via New Orleans and spending a day on the Biloxi beach.

The symposium in Houston did not impress me much. The last day, a Friday, closing the sessions, there was a farewell snack in the Texas Medical Center Library (which I was to visit so many times in the not too distant future). Holding a sandwich and a glass of wine, I met Dr. Hebbel E. Hoff and, in one inspired shot, told him about my misfortunes at Emory. With a broad smile he said: " '*you gonna be here tomorrow morning? If so, come to the Physiology Department at Baylor at 9:00 am sharp*". Like a speeding bullet, my body landed punctually at Dr. Hoff's office that Saturday. He was already there and introduced me to Dr. Mary Brazier (I learned later she was the famous and well-known neurophysiologist, can you imagine my excitement and feelings?). I saw the laboratories, learned about the programs, was also introduced to Dr. Leslie A. Geddes and I was told to send a cv as soon as I arrived back in Atlanta. Everything reminded me of the interview with Prof. Vignaux at the Institute of Radiotechnique years before. Again, my eyes and neurons turned up-side down. Full of excitement, I told the story to Nilda, *blabla-blabing *like a parrot.

The answer did not take long. Dr. Hoff himself called me up with an appealing offer: A position as biophysicist within a contract with National Aeronautic and Space Administration (NASA) to work on some respiratory subjects under the supervision of Lee E. Baker, who was then a Graduate Student and Instructor. Early in May, and after repeating our previous gulf route, but this time pulling a U-Haul trailer full of our stuff, we settled down in Houston, where we rented a duplex apartment in the Hermann Park area, very close to the Medical Center.

It was to be a brief first period at Baylor (only 9 months, unfortunately). In short: The six-week Summer Course in June-July 1962 (studying physiology like hell, reviewing electronics, running experiments every day, interacting with classmates from different origins, working 12 hours or more daily and even finding time to have some fun. Somehow, and in a very rudimentary way, part of that whirling experience was poured in four articles, three of them published in Spanish in Argentina [20-23] in an unknown local journal, which disappeared a long time ago. Childishly, I wanted to let people know what new world I had found, without realizing that, truly nobody really cared much about my accomplishments in this part of the planet. Unexpected to me was that the report [20], after a field trip to the Texas A & M Veterinary School made in July 1962, in College Station, would become the seed of a successful project 10 years later [24,25]. Simultaneously, I was carrying out a bunch of respiratory measurements trying to determine phase impedance changes during respiration. Lee Baker and I produced a confidential report to NASA, obviously not allowed to be published. Interestingly, NASA gave me the clearance to work on that Project. Remember, that was the time of the first American sub-orbital flight, with John Glenn as the first astronaut, who had lateral tranthoracic electrodes to record his respiration using the impedance technique. Yes, that period introduced me to that technique so marking one of the main tracks I was to follow thereafter. Moreover, a suggestion from Dr. Hoff pushed me to register as a Ph.D. graduate student in physiology and I took a few initial courses. No waste that year, indeed.

## *Universidad Nacional del Sur *(UNS) in Bahía Blanca

However, a second big mistake was awaiting me in the bushes. During my visit to Princeton, I had seen an old Argentine friend of mine, a mathematician and lyric tenor (the latter a hobby). A bright guy who died young. He had arranged a return to Argentina with a professorship at the *Universidad Nacional del Sur*, city of Bahía Blanca (South of the Province of Buenos Aires). To make the story short, he put me in contact with another professor in that university (whom incidentally I had met before) and they talked me into signing a three year contract as Associate Professor of Electronics. Yes, I stuck my foot in the wrong hole, again. Things did not work out as expected and soon I desperately wanted to leave the place ... and Nilda was pregnant of our second daughter, Veronica (who was born in Bahía Blanca, in 1963. Later, after spending many years in Brazil and in the USA, she now is settled as investigator in a beautiful research institute in a very small town, Anillaco, La Rioja, Argentina). In the middle of this new collapse, I received an unexpected letter from Leslie A. Geddes inviting me to rejoin the program at Baylor; they had approval for an important grant from NIH (National Institutes of Health). It took sometime to arrange the tons of pending businesses, committments, documents, including my appendix surgery, while the people at Bahía Blanca looked at me with their eyes madly crossed. That place would have been my academic burial if I had stayed there, quite similar to my previous bad experience at Emory. On February 1966, I landed back in Houston and two months later, in April, Nilda arrived with the two girls, Fabiana, 4, and Veronica, 2.

In spite of the weak academic atmosphere surrounding me at UNS, there were people with drive and feeling able to trigger responses. More than that, a poor environment often acts as a stimulus to counter-react and I tend to be pushy when the situation gets tough. I was in charge of two of the electronics courses and that forced me to study a lot. The students were good, always asking for more. We could offer laboratory exercises they had never had before. I got in touch with Dr. Antonio Monteiro, a renowned Portuguese mathematician with whom I studied quite a bit of mathematical logic. In the end, a few articles were published. Four in Spanish in local journals [26-29] and other two in the international arena [30,31]. All in all, considering the periods with Emory and in Bahía Blanca, the overall toll my family and I paid made us lose five years of good productive happy life. **A moral for the young**: **Beware of golden promises, think it over twice, thrice or more before giving an affirmative answer. Time is the most precious asset you have ... and it is irreversible**.

## Biomedical Engineering at Baylor College of Medicine

**Figure 3:**
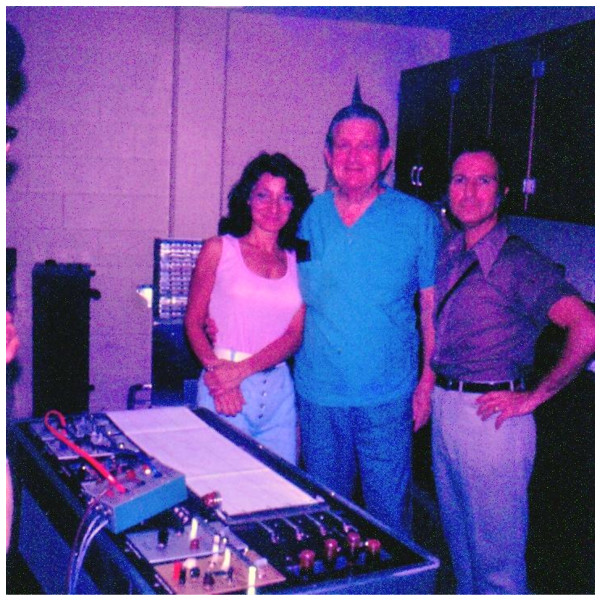
**My old laboratory in the Department of Physiology at Baylor College of Medicine (1973)**. My wife Nilda, Dr. Hoff and Max in front of the three-channel Physiograph (ink recorder) I had. Experiments were run routinely every week for medical and graduate students in the general lab (not this one), where there were several stations, each equipped with the same kind of recorder. Respect and affection to animals was always underlined and practiced. Often, Dr. Hoff said: "Never make an incision longer than you actually need, and do not forget to keep a clean surgical area. That too must have an aesthetic content."

**Figure 4:**
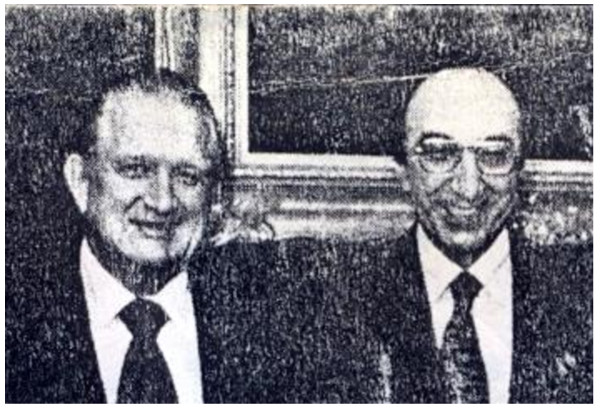
**Hebbel E. Hoff (left) and Michael DeBakey (right), Chairman of the Department of Physiology and President of Baylor College of Medicine, respectively, during the years I was there (1962-3; 1966-1973)**.

Without doubt, this time at Baylor was the happiest and most productive period of my younger life. I finished my doctorate in physiology, lectured in different courses (to medical students, to graduate students), gained enormous experience with experimental techniques working with many species (i.e., frogs, turtles, snakes, lizards, caimans, rabbits, cats, dogs, sheeps, burros, horses, cows), produced an acceptable number of papers and even collected material and ideas for years to come. The atmosphere Les and Hebbel created was like a rich broth, full of nutrients, generous actions, and especially of academic freedom [32,33]. Often Hebbel used to underline his comment: "*Do you enjoy what you do here? If not, I am sure someplace else you'll find what you're looking for." *How true this assertion is, liking our activity, falling in love with it ... not in vain the hypothalamus has the motivation area. Every week there were seminars, sometimes people from other universities came. Some of our teachers were unforgettable, such as Roger Guillemin, who later in 1977 shared the Nobel Prize of Medicine and Physiology for his contributions to neuroendocrinology, and who still honors me with his friendship [34]. Much I have to thank and gratefulness is a sentiment I deeply recognize (Figures 3 and 4).

## *Universidad Nacional de Tucumán *(UNT) and CONICET

Again a slow snail-mail letter from a slightly older friend and former university classmate, planted in me the idea of going back to Argentina for a year or so, this time to the northwest, to the *Universidad Nacional de Tucumán*. There was a small Laboratory of Bioelectronics that had been created a few years before [35]. Once more, we packed up and off we went at a period when wild violence, terrorism and state dark counter-terrorism had taken over Argentina. That year, 1972, I worked like hell surrounded by a small group of electrical engineering students craving for ideas, full of enthusiasm, who represented the best reward I could dream of. I established the first organized bioengineering course in Argentina, "Introduction to Bioengineering", with laboratory exercises using a physiograph that someone had bought earlier. The course ran for four full months, with two three-hour lectures a week plus one full eight hour-day per week devoted to the laboratory. We run essentially all the "Experimental Physiology Laboratory Manual" that was in those days used at Baylor and that had been written by Hoff and Geddes, in a way a projection of Geddes' doctoral dissertation.

**Figure 5:**
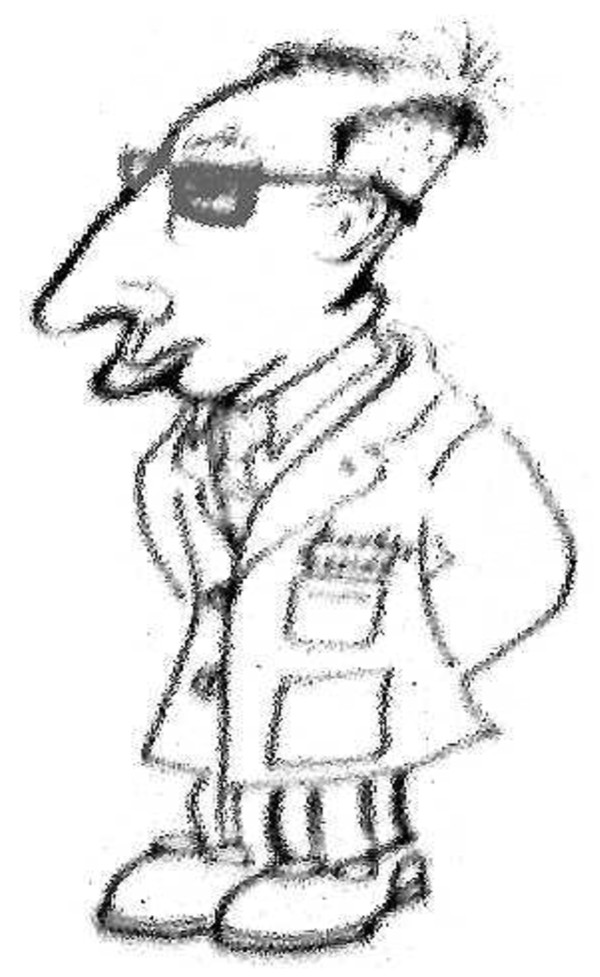
**MEV's caricature**. This is the way a former student of mine, Héctor Hugo Torres (superb draftman), characterized me in 1972, when experiments were carried out surrounded by explosions and riots. Quite an honor. A well depicted nasal appendix clearly synthesizes Max's personality. He gave it to me to manifest his affection and recognition, the best reward a teacher may obtain.

Many are the stories around this short period (10 months only), some funny, as the first thoracotomy on a dog without a mechanical respirator (not available); that function was carried out for several hours by the students, taking turns to blow air in from their very lungs. An atmosphere of work, hope and joy prevailed among my small group of students in spite of the general situation and they always found the happy side of the coin (Figure 5). Other moments were much less funny, as the day we were doing a frog's heart demonstration and a tear gas bomb got into the small lab through a small window. Needless to say, we were forced to run out leaving everything as it was. Outside, all around the main university building where we were, the ERP (*Ejército Revolucionario del Pueblo*) joined by many students and the *Montoneros *group were rioting while the police counter-attacked. We saw on two corners fires sending huge flames upward from tires and wood. The school year was almost over, I was very depressed and did not understand well the sad situation we were going through, even facing family difficulties which included my wife's mother sudden death. Thus, early in January 1973 we returned to Houston where, absolutely uncommon, our reception was with snow. I re-started another short period of only nine months, in spite that "my conviction was to settle there for good." The human being ... or what a psychological mess I was submerged in, troubled by doubts, being torn apart by different feelings.

**Figure 6:**
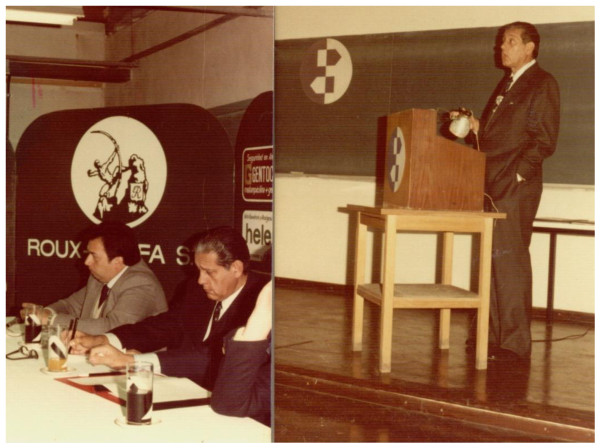
**René G. Favaloro**. He was a well-known cardiac surgeon, developer of the coronary by-pass in the 1960's. He visited the Bioengineering Lab in Tucumán, in 1982. Organized by SABI. Left panel (left to right): Eduardo Valdez, at that time member of the Laboratory, and Dr. Favaloro. Right panel: Favaloro offers a conference.

Still, that year at Baylor meant enormous activity. The last summer course for me, while the overall situation had also changed within the department of physiology, some grants had not been approved and rumors of a different sort upset the usual happy working mood. One day I received a telegram from England, informing me that the IFMBE jointly with the Biological Society of London had awarded me (and collaborators) the Nightingale Prize of Bioengineering, based on a paper published the year before [24,25]. I could not be present during the award ceremony, which was in Germany during the International Congress of the Federation for that year, nor was I in the mood for a trip because the psychological trauma really pervaded me. Meanwhile, letters were pouring in from Argentina, a few asking me to return, others (most of them) advising me to stay where I was ... painfully and trembling, the scale moved to the first choice and this time, yes, it was for good, looking in front of me at an uncertain road. It was September 1973, just when Salvador Allende, in the neighboring Chile, was forced out of power and killed himself in the *Casa de la Moneda*, the Government House in Santiago. Years later, in 1981, I met Dr. René G. Favaloro (Figure 6), who had returned to Argentina in 1971; we became friends and my group collaborated with his for several years in several research projects. One of his comments was: "*What an upheaval moment of the Argentine history when we decided to return to our native country*."

**Figure 7:**
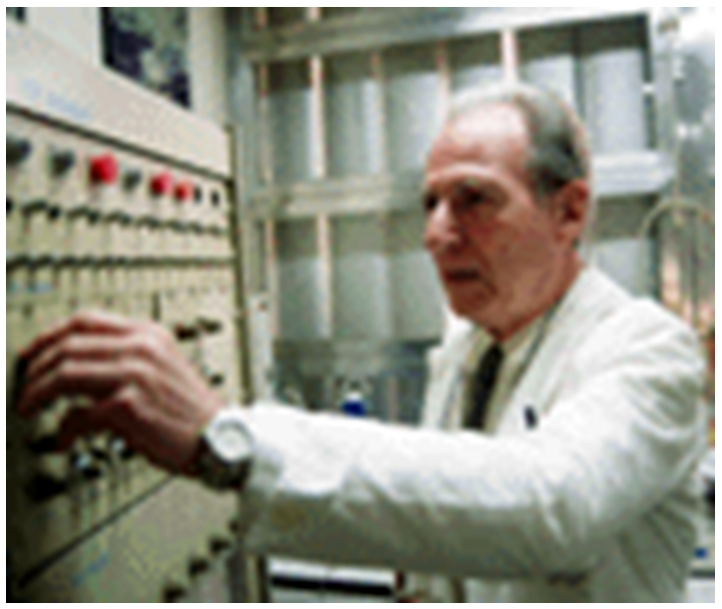
**Max in his Bioengineering Laboratory at the University of Tucumán (UNT) during the 1990's**.

From that year, 1973, until my retirement in 2001, my main activity without interruption, except for short trips always for academic and scientific reasons, was developed at the Bioengineering Laboratory of the *Universidad Nacional de Tucumán*, that is, 28 years, which actually go up to 30 if I count the preliminary 1972 plus an extra year after my retirement that extended me until 2003. This is not the place to report what was done, but work was intense and the laboratory was always busy, with weekly seminars and permanent links with people abroad (Figure 7). There is more detail in a relatively recent long article about bioengineering in Argentina [35]. Aside from some papers, some most significant accomplishments are the establishment of a Research Institute (INSIBIO or *Instituto Superior de Investigaciones Biológicas*), a Laboratory of Bioengineering, an undergraduate program leading to the degree of Biomedical Engineer with the possibility of proceeding to the doctoral level and a National Society of Bioengineering (SABI, *Sociedad Argentina de Bioingeniería*), which already carries 32 active years on its shoulders and 18 conferences. All this reminds me of a basic principle in any academic or scientific project: **first come the people, second the equipment, and finally the buildings**. Changing the order leads to frustrations, often irreparable.

## Retirement and *Universidad de Buenos Aires *(UBA)

In Argentina, retirement is a dreaded stage of life, mostly because it is compulsory (at 67 for men and 65 for women, no matter how good your mental health is or how eager you still are to continue a bit longer), with the exception of those people enjoying privileges (say, political or managing positions, as ministers, legislators, rectors, deans, high ranking directors or the like, who may proceed for several additional years. The other factor causing uneasiness is retirement income, always significantly below a fair level, which those few enjoying the abovementioned privilege do not undergo because the amounts they receive are by far more generous. Such a state of affairs means that very many retirees carry on endless lawsuits (years) against the National Social Security Agency (ANSeS, is the Spanish acronym for it), claiming for what they consider to be the fair amount of money to be paid, in fact, to be returned, for we have contributed in a kind of savings account. The Agency always is in an advantageous position simply because biology, not infrequently, puts the old contending retiree (often well above 80) out of the game and so the lawsuit is suddenly terminated. The obvious consequence is that many retirees search for a job ... not easy to find. The intelectual retiree, however, is not only motivated by monetarial needs, but also because he/she likes his/her job (teaching, research, consultation). Furthermore, it is good for the country, to keep active a group of such experienced people. A concept such as the Council of Elders used to be a highly valued and respected institution among aboriginal and ancient civilizations.

**Figure 8:**
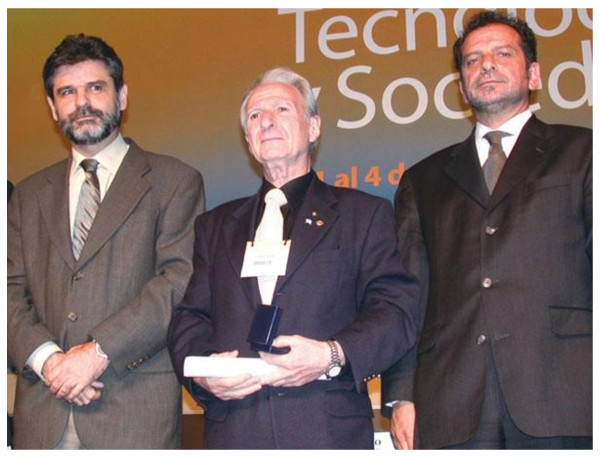
**Podium during award ceremony**. In Buenos Aires, November 2004, when MEV was awarded the Houssay Prize of Science and Technology. At his right, the then Minister of Education, Licenciate Daniel Filmus.

After spending three months in Lima, Perú, at the *Pontificia Universidad Católica del Perú *(PUCP), in 2004, as Honorary Professor, almost two months in Bologna, Italy in 2006, at the Institute of Advanced Studies of the University of Bologna with a Fellowship for Senior Researchers and, from 2004 to 2007 supervising a project on rehabilitation at the *Universidad Nacional de San Juan *(West Argentina), where I held a position of Visiting Professor and directed a National Research Council Fellow (called CONICET in Argentina, or *Consejo Nacional de Investigaciones Científicas y Técnicas*) in her doctoral dissertation, I had the very welcome surprise of being hired by the *Universidad de Buenos Aires *(UBA) in its Institute of Biomedical Engineering, where I am fortunate enough to lead a small research group. So, in the end, I am back to my *Alma Mater*. Finally, during these last years (2004 and 2010), I was able to put forward two books, in a way of gathering information collected over my career [36,37]. More than ten years before, there was another book, this time in collaboration with several authors [38]. In 2004, the Argentine Secretariat of Science and Technology awarded me the Houssay Prize (Figure 8). Overall, no big deal, but anyway, very nice. Thus, since retirement, I have not been idle, for which I humbly thank the Great Engineer, keeping content with the things that I have received.

## Last words and even perhaps a message to the young

After all the previous talk, there may be a few questions popping up in the reader's mind, as a friend of mine suggested,

-Were you and your team able to produce world class research in Argentina?

-Were there any benefits to the world's humanity?

-What frustrations did you have?

-What recommendations can you offer to those investigators who must compete with investigators in the rich countries?

Answers are not easy because a lot of personal feelings become entangled with the experiences collected over many years. Some bias shows up almost as an unavoidable risk, but let me try:

Speaking of "world class research" sounds somewhat presumptous; such qualifation should be given by those who found useful or inspiring content in my work. Production in quantitative terms there was, along with an aceptable number of citations, if we are to follow the current evaluation criteria. Although every sensible researcher knows that only time offers a more definite statement. Quality should weigh more than quantity. For the time being, I dare say I am satisfied and hope that my former collaborators are too. Specific contributions, as another good friend asked me, can include the measurement of myocardial impedance during fibrillation to dose the applied recovery discharge, the use of intraventricular pressure-impedance diagram to obtain cardiac efficiency, the application of milk bulk impedance to estimate bacterial content, or resurrection of discrete deconvolution analysis that showed good results in evaluating the renal retention function. Reference [35] collects most of the references that deal with these subjects.

Benefits ... perhaps, those contributions mentioned above are available and I hope someone found them useful, however, if not, we had better remember that before anything else, we are simply men and women, that being is much better than having and that independently of how much richness, or power, or knowledge, or worldly glories we might collect and store, the really important and significant measure is, and will be, how much we love and how much we have loved, and remember that the scientific endeavor calls for a lot of love. Is this an adequate answer?

Frustrations abounded, yes, and many of them. Dreaming always of a well-paid team of dedicated collaborators, many young eager researchers, surrounded by good and sufficient equipment located in a comfortable building, where work, musings, and exchange of ideas are carried out as smoothly as bacteria proliferating within a nutricious and plentiful culture broth. Far were we from such an ideal situation. However, difficulties, honest poverty, and shortages often act as stimulants and, in a way, such phenomenon took place ... or at least that is what I want to believe. The Atlanta, Bahía Blanca experiences and later on the overall Argentine situation during the dictatorship years, when in Tucumán, represent good examples. In the first place I was really alone, not fully in Bahía though, and followed and supported by a group of young students and a few hard-minded colleagues in the latter.

Recommendations encompass responsability, as for example advising a young person to stay home when he/she boldly declares an overt wish of emigrating because "here there is no way out or there is no chance here." He/she may later confront me with anger, as being the source of his/her failure to leave the country and succeed in science. Anyway, there are a few additional thoughts to mention,

-first: it is a highly personal decision that only he/she can take;

-second: spending two or three or more years abroad, in a good laboratory under the direction of a recognized scientist and teacher is no doubt a terrific experience, usually supplying an imprint that becomes a kind of acquired DNA. However, and going back to my two significant mistakes, I should add: think it over many times and balance out the pros and cons before giving a yes.

-third: going back to the native country is even a more personal decision than the original decision, when the researcher chose to leave, but by far more complicated. A major consideration here is he/she is older and developed attachments to the surrogate country, because in all likelihood there is also now a family involved;

-four: in case the decision turns toward the return ... well, get ready, breathe deeply and dive in with all your guts and power. The struggle will be hard and there is no guarantee, but always enjoy what you do, if you don't, go someplace else. Besides, you are paid to do what you supposedly like to do-research and development.

Good point to add: Many times I have been asked why I came back to Argentina. The answer is straight and simple, but it took me decades to come down to terms with myself and realize, because as said above: "*es ist mein Haus, und ich liebe es sehr*."

And what about BME? Is it a good choice for the young? Yes, absolutely yes ... This century will produce outstanding and breaking-through new contributions, perhaps not even dreamt of!

Looking up to the skies above, to the mountains in front of me, to the sea beyond, I now take into account once more that no matter how much you, we, I, may have collected, -money, power, fame or knowledge-, the most important and significant question reduces to a simple *how much have we loved*. Boy ... we may run into trouble when trying to faithfully answer such a question.
